# Severity of migraine-like symptoms and its impact on executive functions in university students: a mediation model analysis

**DOI:** 10.1017/S1463423625100649

**Published:** 2025-12-02

**Authors:** Md Dilshad Manzar, Mohammed F. Salahuddin, Faizan Kashoo, Dejen Nureye, Wakuma Wakene Jifar, Seithikurippu Pandi-Perumal, Ahmed S. BaHammam

**Affiliations:** 1 Department of Primary Nursing Care, College of Nursing, Majmaah University, Majmaah 11952, Saudi Arabia; 2 Department of Pharmaceutical Sciences, School of Pharmacy & Health Professions, Notre Dame of Maryland University, Baltimore, MD 21210, USA; 3 Department of Physical Therapy and Health Rehabilitation, College of Applied Medical Sciences, Majmaah University, Al Majmaah 11952, Saudi Arabia; 4 School of Pharmacy, College of Medicine and Health Sciences, Mizan-Tepi University, Mizan-Aman, Southwest Ethiopia Peoples’ Region, Ethiopia; 5 Research Unit of Neuro-Inflammatory and Cardiovascular Pharmacology, Faculty of Science, University of Dschang, P.O. Box 67, Dschang, OR, Cameroon; 6 Department of Pharmacy, College of Health Sciences, Mettu University, Mettu, OR, Ethiopia; 7 Centre for Research and Development, Chandigarh University, Mohali 140413, PB, India; 8 Division of Research and Development, Lovely Professional University, Phagwara 144411, PB, India; 9 University Sleep Disorders Center, Department of Medicine, College of Medicine, King Saud University, Riyadh, Saudi Arabia; 10 Sleep Disorders Center, King Saud University Medical City, Riyadh, Saudi Arabia; 11 National Plan for Science and Technology, College of Medicine, King Saud University, Riyadh, Saudi Arabia

**Keywords:** attention, fluid intelligence, memory, migraine, planning, reasoning

## Abstract

**Background:**

The influence of severity of migraine-like symptoms on different levels of executive functions is not well established. In this study, we investigate the impact of severity of migraine-like symptoms on the relationship between core-level executive functions (attention and memory) and fluid intelligence.

**Methods:**

A cross-sectional study was conducted on university students (n = 427, age = 20.7 + 1.8 years). Participants completed self-report measures of Migraine Screen Questionnaire (MS-Q), single-item visual analogue scales (VASs) each for the subjective accounts of problems in core-level executive functions (attention and memory), and a single-item VAS for problems in fluid intelligence (PFI), and sociodemographics tool. The mediation effect model was used to determine the relationship.

**Results:**

The study found a correlation between i) attention problems and severity of migraine-like symptoms (b = 0.109, standard error (SE) = 0.026, p < 0.001), ii) severity of migraine-like symptoms and memory problems (b = 0.318, SE = 0.076, p < 0.001), and iii) severity of migraine-like symptoms – PFI (b = 0.243, SE = 0.083, p < 0.003), with an indirect effect of attention problems on memory problems and PFI and no correlation between severity of migraine-like symptoms and PFI.

**Conclusions:**

Self-reported accounts of problems in core-level executive functions and fluid intelligence are correlated. Severity of migraine-like symptoms may mediate the inter-relationship between some core-level and higher-level executive functions.

## Introduction

The prevalence of migraine is currently estimated to be between 14% and 15% worldwide, and it is responsible for a substantial global burden of years lived with disability (Steiner and Stovner, 2023). The disability-adjusted life years for migraine have increased by more than 58% globally in the years 1990–2021 (Dong *et al.*, 2025). It is frequently associated with impaired social and professional lives and decreased productivity (Agosti, 2018; Buse *et al.*, 2009). Multiple factors, such as mental stress, noise, exhaustion, dieting, insomnia, and alcohol consumption, are associated with the onset of migraine (May and Schulte, 2016). Young adults, especially university students, are more susceptible to migraines than other age groups due to the prevalence of migraine triggers, such as educational stress and irregular sleep patterns (Anaya *et al.*, 2022). It is estimated that 12%–23% of university students worldwide suffer from migraines (Flynn *et al.*, 2023); such a higher occurrence of migraines may impair students’ academic performance and quality of life (Bigal *et al.*, 2001).

A host of hierarchic cognitive processes that help concentrate instead of relying on instinct or intuition are known as executive functions (Burgess and Simons, 2005). Executive function is organized into a set of core and higher-order cognitive processes – including inhibition, working memory, and cognitive flexibility – that enable goal-directed behaviour and support reasoning, planning, and problem-solving (fluid intelligence). Competencies in executive functions are necessary for good health with a conditional relation for both physical and mental aspects (Diamond, 2013). Moreover, these competencies are important for psychosocial development and help in achieving success in school and in life (Diamond, 2013). Executive functions have a hierarchical classification with core-level and higher-order executive functions. The core-level executive functions comprise inhibition, working memory, and cognitive flexibility (Lehto *et al.,* 2003; Diamond, 2013). Attention is a type of interference control, which is a component of the inhibition – a core-level executive function (Diamond, 2013). The core-level executive functions coordinate to construct higher-level executive functions known as fluid intelligence (Collins and Koechlin, 2012; Diamond, 2013). The aptitude to reason, solve problems, and recognize patterns is fluid intelligence (Ferrer *et al.,* 2009; Collins and Koechlin, 2012).

Executive function impairment contributes substantially to migraine-related disability (Gil-Gouveia and Martins, 2019). Migraine is associated with executive functions deficits such as attention deficit (Faedda *et al.*, 2017), executive dysfunction (Francesco Le Pira *et al.*, 2014), immediate and delayed memory impairment (Braganza *et al.*, 2022), and slower information processing speed (Riva *et al.*, 2006). Evidence shows that people with migraine have deficit in fluid intelligence such as poor problem-solving and decision-making skills (Vuralli *et al.*, 2018; Mongini *et al.*, 2005). Most of the studies investigating migraine–executive functions relationships have tried to view this aspect with the narrowed prism of association between migraine and a specific executive function (Russo *et al.*, 2022; Vuralli *et al.*, 2018). The impact of migraine on the hierarchy of executive functions has not been thoroughly studied. Given this gap in the literature, the present study aims to explore the dynamics of migraine and executive function by assessing the impact of severity of migraine-like symptoms on the relationship between core-level executive functions (attention and memory) and fluid intelligence. This research question is significant, as it could provide a more comprehensive understanding of the interplay between migraine and executive functions, which could potentially inform more effective interventions for individuals suffering from migraines.

We hypothesize that migraine mediates the relationship between core-level and higher-level executive functions. This hypothesis is based on the premise that migraine, as a neurological condition, could potentially disrupt the cognitive processes involved in executive functions. If confirmed, this hypothesis could contribute to the existing body of knowledge by providing empirical evidence on the mediating role of migraine in the relationship between core-level and higher-level executive functions. Therefore, in this study, we explored migraine–executive function dynamics by assessing the impact of severity of migraine-like symptoms on the relationship between core-level executive functions (attention and memory) and fluid intelligence.

## Material and methods

### Participants and procedure

In this study conducted from December 2020 to March 2021, the target population was university students, and the accessible population was students of Mizan-Tepi University (MTU), Mizan-Aman city branch, Mizan-Aman, Ethiopia. The study sample was selected using simple random sampling from the sampling frame, that is, the list of enrolled students at MTU. The selected students were contacted physically in person. A sample of 600 students was earmarked, of which 427 students (age: 20.73 ± 1.84 years) completed this study. All students enrolled in the regular courses of the university were included. Students under 18 years of age were excluded to prevent difficulties in gaining parental consent, as many of their parents may reside in remote locations. The researchers explained the objectives and methods of the study in simple language to students. Students were then provided with a survey package comprising a plain-language overview of the study’s objectives, a consent form (to participate and publish), and questionnaire instruments. All participants signed the informed consent forms. The participating students were informed about their right to withdraw without fear/apprehension of grades at any time; there were no incentives, no risks, and a strict process to maintain the confidentiality of personal information. Further, participants were provided with a contact person’s details to communicate their queries, doubts, and suggestions.

The study was done following the procedures in the Helsinki Declaration of 2002, together with the World Medical Association General Assembly amendments, Fortaleza, Brazil, 2013. The Ethics Committee at the College of Medicine and Health Sciences, Mizan-Tepi University, Mizan-Aman, Ethiopia, approved the research procedure.

The survey questionnaire contained (i) a Migraine Screen Questionnaire (MS-Q), (ii) single-item visual analogue scales (VASs) each for the subjective accounts of attention problems, memory problems, and problems in fluid intelligence (PFI), and (iii) sociodemographics. The survey packet and questionnaire were in English because MTU is a federal university teaching in English. Thus, students are English-proficient.

### Measures

#### Migraine Screen Questionnaire

Lainez and colleagues developed this brief tool (MS-Q) to screen migraine at the Neurology Department of Universidad de Valencia (M. J. Láinez *et al.*, 2010; M. J. A. Láinez *et al.*, 2005; M D Manzar *et al.*, 2020). The MS-Q has five items scored as 0 for no and 1 for yes responses. The MS-Q has been commonly used in both clinical and research settings and was developed following the International Headache Society (IHS) criteria for diagnosing migraine (M. J. A. Láinez *et al.*, 2005; Olesen and Lipton, 1994). Individual item scores are added to generate a total score, and a cut-off score of 4 and above is used to screen migraine cases (M. J. Láinez *et al.*, 2010; M. J. A. Láinez *et al.*, 2005; M D Manzar *et al.*, 2020). The tool has a sensitivity of 93% and specificity of 81% for screening migraine at a cut-off score of 4 and above (M. J. A. Láinez *et al.*, 2005). The MS-Q has been found to have psychometric validity in migraine patients, primary care settings, and university students (M. J. Láinez *et al.*, 2010; M. J. A. Láinez *et al.*, 2005; MD Manzar *et al.*, 2020).

#### VASs for measuring subjective complaints of problems in attention, memory, and PFI

Three separate single-item VASs (length: 100 nm scale; score range: 0 to 10) were used to record responses regarding subjective complaints of problems in attention, memory, and fluid intelligence by the university students. In all these scales, higher scores were indicative of an increasing level of problems in attention, memory, and fluid intelligence. ‘Do you have difficulties paying attention? (e.g., to a conversation, a book, or a movie)’ was used to assess problems in attention. ‘Do you experience frequent memory loss? Please rate on the scale below: Do you forget the occurrence of events, even the more recent ones, appointments, etc.?’ was used to assess problems in memory. ‘Do you feel that you are slower when reasoning, planning activities, or solving problems?’ was used to assess problems in PFI. VASs employed simple language and clear terminology to improve participants’ comprehension, reduce the risk of misinterpretation, and enhance the accuracy of responses. Similar VASs have been used in previous works to measure attention complaints in university students (M D Manzar *et al.*, 2020). In this study, the severity level of problems in attention, memory, and PFI was based on a cut-off score of 6 and above in the corresponding VAS (Manzar *et al.*, 2020). A score of 0 to 5 indicated no to mild levels of problems in attention, memory, and PFI. While a score of 6 to 10 implied a moderate to severe level of problems in attention, memory, and PFI. Similar cut-off scores have been explored and found to have adequate validity for single-item VAS (length: 100 nm scale; score range: 0 to 10) in screening diverse clinical conditions such as pruritus, attention complaints, and dental anxiety (Appukuttan *et al.*, 2014; Kido-Nakahara *et al.*, 2015; M D Manzar *et al.*, 2020; Reich *et al.*, 2012).

### Statistical analysis

SPSS version 23.0 (SPSS Inc., Chicago, IL, USA) was used to perform the descriptive analysis. Additionally, the PROCESS macros version 4.0 for SPSS was used to implement the mediation model analysis (Hayes, 2013; Hayes and Coutts, 2020). There were no person-level missing values in the study sample (n = 427).

#### Handling of missing data

In our study sample, 37% of cases had at least one missing value, accounting for 8.9% of the data points. These missing values were random with no trends. Therefore, we employed a multiple imputation method with five iterations before performing the mediation model analysis.

PROCESS 4.0 does not have features to manage mediation analysis using pooled imputed datasets. This was resolved pragmatically using the fifth iteration imputed dataset in the mediation analysis.

#### Mediation analysis procedure

We used the mediation analysis procedure proposed by Baron and Kelly, 1986, along with modifications suggested (Baron and Kenny, 1986; Zhao *et al.*, 2010). The presence of a significant indirect effect was estimated by the absence of zero from the adjusted bootstrapped confidence interval (CI) for the unstandardized coefficients (Preacher and Hayes, 2004).

In all, three separate mediation models were analysed (i) predictor: level of attention problems, dependent variable: level of memory problems, and mediator: severity of migraine-like symptoms (MS-Q score), (ii) predictor: level of attention problems, dependent variable: PFI, and mediator: severity of migraine-like symptoms (MS-Q score), and (iii) predictor: level of memory problems, dependent variable: PFI, and mediator: severity of migraine-like symptoms (MS-Q score). Age was used as a covariate in all three mediation models.

#### Adjustment for multiple testing

To manage the prospect of inflated type I error due to multiple testing, we adjusted the significance level (alpha) to p = 0.017 (0.05/3, where three is the number of mediation models/comparisons). Furthermore, the CI of the bootstrapped coefficients was changed to 98.33.

#### Replicability of coefficients

To secure the replicability of the coefficients in the mediation models, we edited the PROCESS syntax to fix seeding at 5026. This is important because working with bootstrap sampling can give slightly different coefficients every time the test is repeated.

## Results

### Participants’ characteristics

The average age of the participating students was 20.73 ± 1.84 years, with the majority being males (69.3%). The students’ scores on the VAS for attention problems, memory problems, and PFI were 3.52 ± 3.02, 3.35 ± 2.69, and 3.73 ± 2.95, respectively. The average score on the MS-Q was 1.58 ± 1.67 (Table 1).

**Table 1.** Participants’ characteristics of university students

Characteristics	Mean ± SD/frequency (percentage)
Age	20.73 ± 1.84
Gender	
Female	114 (26.7)
Male	296 (69.3)
Did not report	17 (4.0)
Attention problem	
VAS scale score (on a scale of 0 to 10)	3.52 ± 3.02
Attention problem severity	
Moderate to severe level of attention problems	77 (18.0)
No to mild level of attention problems	310 (72.6)
Did not report	40 (9.4)
Memory problem (on a scale of 0 to 10)	
VAS scale score (on a scale of 0 to 10)	3.35 ± 2.69
Memory problem severity	
Moderate to severe level of attention problems	61 (14.3)
No to mild level of attention problems	330 (77.3)
Did not report	36 (8.4)
PFI (on a scale of 0 to 10)	
VAS scale score (on a scale of 0 to 10)	3.73 ± 2.95
PFI severity	
Moderate to severe level of attention problems	75 (17.6)
No to mild level of attention problems	314 (73.5)
Did not report	38 (8.9)
Migraine severity	
MS-Q score	1.58 ± 1.67
Migraine symptoms^*^	
Yes	62 (14.5)
No	315 (73.8)
Did not answer at least one item of the MS-Q, and the total MS-Q score did not reach 4 from the available responses.	50 (11.7)

* Based on a cut-off score of ≥ 4 for the MS-Q total score; SD: standard deviation.

MS-Q: Migraine Screen Questionnaire; VASs: visual analogue scales of 100 nm were employed to measure subjective accounts of attention problems, memory problems, and problems in fluid intelligence (PFI).

The severity level of problems in attention, memory, and PFI was based on a cut-off score of 6 and above in the corresponding VAS (Manzar *et al.*, 2020). A score of 0 to 5 indicated no to mild levels of problems in attention, memory, and PFI. While a score of 6 to 10 implied a moderate to severe level of problems in attention, memory, and PFI.

The prevalence of migraine symptoms among the participating university students was 14.5%. The prevalence of moderate to severe levels of subjective problems in attention, memory, and fluid intelligence was 18.0%, 14.3%, and 17.6%, respectively.

### Severity of migraine-like symptoms and its impact on attention and memory

**Figure 1.**
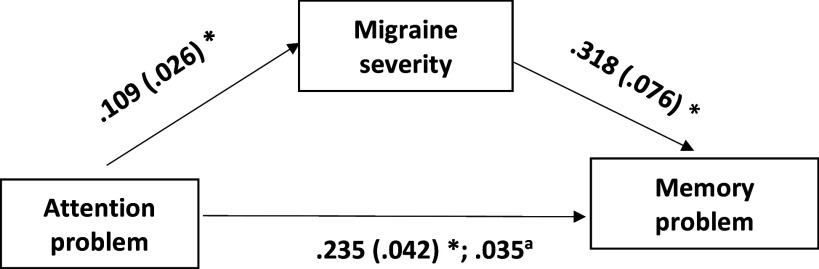
The model with migraine severity (severity of migraine-like symptoms) (MS-Q score) as a mediator in the effect of attention problems on memory problems. Age (in years) was used as a covariate but did not have any significant association (at adjusted p < 0.017). Severity of migraine-like symptoms was assessed by MS-Q score. Attention problems and memory problems were assessed by visual analogue scales to assess subjective accounts of attention and memory-related problems. MS-Q: Migraine Screen Questionnaire. Note. The boxes represent the variables, and the single-headed arrows show the direction of the linear relationship, with the dependent variable towards the arrowheads and the independent variable towards the tail of the arrows. The first values are the unstandardized coefficients, and the second values within brackets are the standard errors; *p < 0.001. ^a^ The indirect effect of attention problems on memory problems through migraine severity was significant (98.33% confidence interval 0.035 [0.011, 0.067]).

Level of attention deficit was closely linked to the severity of migraine-like symptoms (b = 0.109, standard error [SE] = 0.026, p < 0.001) (Table 2; Figure 1). Additionally, a notable correlation was found between the severity of migraine-like symptoms and memory issues (b = 0.318, SE = 0.076, p < 0.001; Table 2, Figure 1).

**Table 2.** Mediating role of migraine severity on the relationship between attention problems and memory problems

Independent variable	Outcome variable	*β*	*b*	SE	98.33% bootstrapping CI		p-Value
					LL	UL	
Attention problem	Migraine severity	0.20	0.109	0.026	0.047	0.172	< 0.001
Severity of migraine-like symptoms	Memory problem	0.261	0.318	0.076	0.134	0.501	< 0.001
Attention problem (direct effect)	Memory problem	0.193	0.235	0.042	0.135	0.336	< 0.001

			98.33% bootstrapping CI				
Types of effect	*b*	SE	LL		UL		p-Value
Total effect	0.270	0.042	0.170		0.370		< 0.001
Indirect effect	0.035	0.012	0.011		0.067		–

MS-Q: Migraine Screen Questionnaire; LL: lower limit; UL: upper limit; SE: standard error; *β*: standardized coefficients; *b*: unstandardized coefficients; CI: confidence interval.

Age (in years) was used as a covariate but did not have any significant association (at adjusted p < 0.017). Severity of migraine-like symptoms was assessed by MS-Q score, and attention problems and memory problems were assessed by visual analogue scales to assess subjective accounts of attention and memory-related problems.

Furthermore, we observed a significant direct effect of attention deficits on memory problems, even after accounting for the severity of migraine-like symptoms (b = 0.235, SE = 0.042, p < 0.001; Table 2; Figure 1). The mediating role of severity of migraine-like symptoms in this attention–memory problem relationship was confirmed through a significant indirect effect, with a 99% CI of 0.035 [0.011, 0.067; Table 2; Figure 1].

### Severity of migraine-like symptoms as a mediator in the attention–PFI relationship

**Figure 2.**
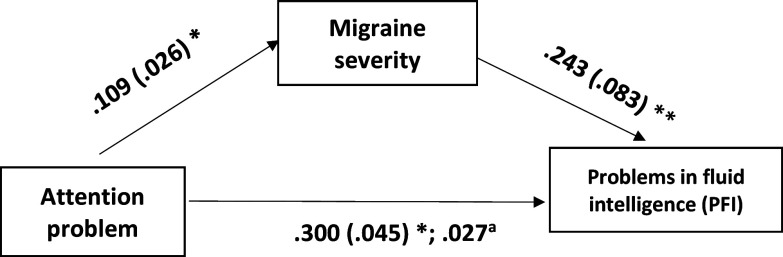
The model with migraine severity (severity of migraine-like symptoms) (MS-Q score) as a mediator in the effect of attention problems on problems in fluid intelligence (PFI). Age (in years) was used as a covariate but did not have any significant association (at adjusted p < 0.017). Severity of migraine-like symptoms was assessed by the MS-Q score. Attention problems and PFI were assessed by visual analogue scales to assess subjective accounts of attention and PFI. MS-Q: Migraine Screen Questionnaire. Note. The boxes represent the variables, and the single-headed arrows show the direction of linear relationships, with the dependent variable towards the arrowheads and the independent variable towards the tail of the arrows. The first values are the unstandardized coefficients, and the second values within brackets are the standard errors; *p < 0.001; **p = 0.003. ^a^The indirect effect of attention problems on PFI through migraine severity was significant (98.33% confidence interval 0.027 [0.005, 0.060]).

Severity of migraine-like symptoms also emerged as a significant factor related to PFI (b = 0.243, SE = 0.083, p < 0.003; Table 3; Figure 2), suggesting that students with more severe migraines tend to report higher levels of PFI. The direct impact of attention deficits on PFI remained significant even when controlling for the severity of migraine-like symptoms (b = 0.300, SE = 0.045, p < 0.001; Table 3; Figure 2). Moreover, a significant indirect effect of attention deficits on PFI was noted: 99% CI = 0.027 [0.005, 0.060; Table 3; Figure 2]. This finding indicates that the severity of migraine-like symptoms significantly mediates the relationship between attention deficits and PFI.

**Table 3.** Mediating role of migraine on the relationship between attention problems and problems in fluid intelligence (PFI)

Independent variable	Outcome variable	*β*	*b*	SE	98.33% bootstrapping CI		p-Value
					LL	UL	
Attention problem	Migraine severity	0.200	0.109	0.026	0.047	0.172	< 0.001
Severity of migraine-like symptoms	PFI	0.136	0.243	0.083	0.044	0.442	0.003
Attention problem (direct effect)	PFI	0.306	0.300	0.045	0.192	0.409	< 0.001

			98.33% bootstrapping CI				
Types of effect	*b*	SE	LL		UL		p-Value
Total effect	0.327	0.045	0.220		0.434		< 0.001
Indirect effect	0.027	0.011	0.005		0.060		–

MS-Q: Migraine Screen Questionnaire; LL: lower limit; UL: upper limit; SE: standard error; *β*: standardized coefficients; *b*: unstandardized coefficients; CI: confidence interval.

Age (in years) was used as a covariate but did not have any significant association (at adjusted p < 0.017). Severity of migraine-like symptoms was assessed by MS-Q score, and attention problems and general cognition problems were assessed by visual analogue scales to assess subjective accounts of attention and PFI.

### Severity of migraine-like symptoms and memory problems: lack of mediation in PFI

**Figure 3.**
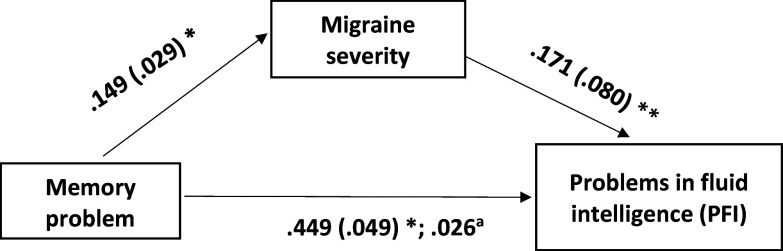
The model with migraine severity (severity of migraine-like symptoms) (MS-Q score) as a mediator in the effect of memory problems on problems in fluid intelligence (PFI). Age (in years) was used as a covariate but did not have any significant association (at adjusted p < 0.017). Migraine severity was assessed by the MS-Q score. Memory problems and PFI were assessed by visual analogue scales to assess subjective accounts of memory problems and PFI. MS-Q: Migraine Screen Questionnaire. Note. The boxes represent the variables, and the single-headed arrows show the direction of the linear relationship, with the dependent variable towards the arrowheads and the independent variable towards the tail of the arrows. The first values are the unstandardized coefficients, and the second values within brackets are the standard errors; *p < 0.001; **p = 0.033 (not significant at adjusted p < 0.017). ^a^The indirect effect of memory problems on PFI through migraine severity was not significant (98.33% confidence interval 0.026 [–0.004, 0.061]).

Contrastingly, while memory problems were significantly associated with the severity of migraine-like symptoms (b = 0.149, SE = 0.029, p < 0.001; Table 4; Figure 3), this did not translate into a mediating effect on PFI. Although students with more severe memory issues often had more severe migraines, this did not significantly correlate with their PFI levels (b = 0.171, SE = 0.08, p = 0.033; not significant at an adjusted p < 0.017; Table 4; Figure 3). The direct influence of memory problems on PFI was, however, significant, independent of the severity of migraine-like symptoms (b = 0.449, SE = 0.049, p < 0.001; Table 4; Figure 3). This suggests that the presence of memory problems in students is likely to contribute to higher PFI, but this relationship is not mediated by the severity of migraine-like symptoms.

**Table 4.** Mediating role of migraine on the relationship between memory problems and problems in fluid intelligence (PFI)

Independent variable	Outcome variable	*β*	*b*	SE	98.33% bootstrapping CI		p-Value
					LL	UL	
Memory problem	Migraine severity	0.246	0.149	0.029	0.080	0.218	< 0.001
Severity of migraine-like symptoms	PFI	0.096	0.171	0.080	–0.021	0.364	0.033
Memory problem (direct effect)	PFI	0.413	0.449	0.049	0.332	0.566	< 0.001

			98.33% bootstrapping CI				p-Value
Types of effect	*b*	SE	LL		UL		
Total effect	0.475	0.047	0.361		0.588		< 0.001
Indirect effect	0.026	0.013	–0.004		0.061		ns

MS-Q: Migraine Screen Questionnaire; LL: lower limit; UL: upper limit; SE: standard error; *β*: standardized coefficients; *b*: unstandardized coefficients; CI: confidence interval; ns: not significant.

Age (in years) was used as a covariate but did not have any significant association (at adjusted p < 0.017). Severity of migraine-like symptoms was assessed by MS-Q score, and attention problems and memory problems were assessed by visual analogue scales to assess subjective accounts of memory problems and PFI.

## Discussion

Our study found a high prevalence of self-reported migraine symptoms and complaints in core-level executive functions such as attention, memory, and PFI. In addition, in this study, the severity of migraine-like symptoms was significantly correlated with problems in both hierarchical levels of executive functions. Vuralli *et al.* (2018) also found similar evidence in their comprehensive review to summarize cognitive dysfunctions in migraine (Vuralli *et al.*, 2018). Our study found a high prevalence of self-reported migraine symptoms and complaints in core-level executive functions such as attention, memory, and PFI among university students. This suggests that the severity of migraine-like symptoms may be associated with hierarchical levels of executive functions, which may, in turn, be associated with adverse academic performance and quality of life in this population. Furthermore, we found that students with memory problems were likely to have PFI, irrespective of their severity of migraine-like symptoms. This finding implies that interventions aimed at improving memory function could potentially enhance fluid intelligence in university students, even in the absence of migraine.

To the best of our knowledge, this study is the first to demonstrate that the increasing severity of self-reported migraine symptoms was comorbid with (i) problems in attention and memory and (ii) PFI in university-attending young adults. However, students with memory problems were likely to have PFI unrelated to severity of migraine-like symptoms. In this study, age did not significantly influence the relationship between memory problems and PFI. The possible reason is that the study sample in our study was college students of similar ages. However, other studies comparing young adults to elderly adults found cognitive decline with increasing age (Kong *et al.*, 2022). Our findings suggest that severity of migraine-like symptoms may play a role in the relationship between certain executive functions and fluid intelligence. However, more research is needed to fully understand these relationships and their implications for individuals with migraines.

The prevalence of migraine in the study sample (14.5%) was lower than the global pooled prevalence rate (18.8%) in university students, and it was slightly lower in comparison with the pooled prevalence of migraine in African university students (15.2%) (Flynn *et al.*, 2023). While it is a common strategy to compare prevalence across different studies, it is better to be informed that there are methodological differences in screening migraine as often across studies (Flynn *et al.*, 2023). Though the prevalence of migraine is relatively lower in African students compared to their counterparts in America, Latin America, Australia, and Europe, the rate is still substantial enough to press the need for exploration of management approaches to minimize the cost of burden and associated outcomes (Flynn *et al.*, 2023). The prevalence of attention complaints (moderate–severe level) was higher in comparison to British university students (12.6%) (Elisa *et al.*, 2016). Few studies have explored the prevalence of subjective memory problems in university students or young people. In the present study, the prevalence of subjective memory problems (14.3%; moderate–severe level) was very high (more than double) in comparison to that reported in a sample of British young adults (5.5 to 6.3%) (Begum *et al.*, 2014).

Patients with migraine present with low serotonin (5-hydroxytryptamine [5-HT]) levels (Comings, 1994; SICUTERI, 1972). 5-HT released from the periaqueductal grey (midbrain) modulates the descending pain pathway by increasing opioid-mediated enkephalin release, inhibiting pain signal transmission to the secondary neuron (Ossipov *et al.*, 2014). Low serotonin levels are implicated in migraine pathogenesis via disinhibiting release of calcitonin gene-related peptide (potent vasodilator) (Aggarwal *et al.*, 2012), and this hypothesis has been further corroborated when 5-HT agonists like triptans alleviate migraine headaches (Clemow *et al.*, 2020; Negro *et al.*, 2018). On the other hand, low serotonin levels are implicated in cognitive deficits (like attention, decision-making, problem-solving, and judgement), and restoration of 5-HT activity may have beneficial effects (Švob Štrac *et al.*, 2016). Chronic administration of valproic acid increased brain levels of serotonin, among other neurotransmitters, in the hippocampus, resulting in an improvement in cognitive functioning (Acosta *et al.*, 1996). Moreover, serotonin and norepinephrine reuptake inhibitors, such as venlafaxine and duloxetine, have been suggested to have neuroprotective activity (Zhang *et al.*, 2019) such as improved performance of working memory (Lauterbach *et al.*, 2010) and attentional deficits (Russo *et al.*, 2022). Thus, the current study speculates that low serotonin levels may increase severity of migraine-like symptoms and executive function deficits.

Our study further contributes to the extant literature that students with increased severity of migraine-like symptoms (ought to have low 5-HT) and attention problems (ought to have low 5-HT) are prone to increased risk of developing memory deficits and PFI. In the current study, students with severe migraine and attention problems were more likely to have severe memory problems. The finding is supported by a study conducted among 26 456 adults that reported migraine and attention problems are comorbid (Hansen *et al.*, 2018). A recent meta-analysis of eight studies found that migraine and attention-deficit hyperactivity disorder (ADHD) are more likely to occur simultaneously (Salem *et al.*, 2018). Similarly, a study reported that the visuospatial and verbal memory abilities of 14 patients with migraine were significantly decreased compared to those of the control group (Le Pira *et al.*, 2000).

Though it is well established that migraine is associated with both attention and memory complaints (Begasse de Dhaem and Robbins, 2022), to the best of our information, the present study is the first one to show that increasing severity of migraine mediates the relationship between perceived attention deficit and memory problems in a sample of university-attending young students.

In the present study, severity of migraine-like symptoms mediated the relationship between perceived attention problems and PFI. Few studies have explored the relationship between perceived attention deficits and PFI in the context of migraine. Extensive narrative reviews on cognitive dysfunctions in migraine have collected evidence from different studies to show that all three variables, that is, migraine, attention deficit, and PFI, are related (Vuralli *et al.*, 2018). Mongini *et al.*, 2005 showed that patients with migraine have an impaired ability to solve problems and make decisions; both are aspects of PFI (Mongini *et al.*, 2005; Vuralli *et al.*, 2018). Furthermore, Vuralli et al summarized that migraine is associated with decreased PFI, more specifically, impaired verbal reasoning, (future)-planning, and problem-solving ability (Vuralli *et al.*, 2018). However, few original studies have explored the relationship between these three variables in a single model. To the best of our information, the current study is the first to show that increasing severity of migraine mediates the relationship between perceived attention deficit and PFI in a sample of university-attending students.

In the current study, memory complaints were associated with PFI and severity of migraine-like symptoms. However, severity of migraine-like symptoms did not mediate the relationship between memory complaints and PFI. This suggests that students with memory problems were likely to have PFI, such as problems in reasoning, planning, and problem-solving unrelated to severity of migraine-like symptoms.

### Limitation

However, our study has several limitations that should be considered. First, our findings are based on self-reported measures, which may be subject to bias. Though the study participants were given a plain-language summary of the study, VASs used simple language to enhance comprehension and response accuracy and provided contact information for seeking clarification for their doubts. However, there is still a possibility of information bias due to participants’ misinterpretation or misunderstanding of questionnaire items, which may have influenced the accuracy of their responses. Future studies could benefit from using objective measures of executive functions and severity of migraine-like symptoms. Second, our study was cross-sectional in nature, which prevents us from making causal inferences. Longitudinal studies are needed to confirm the relationships observed in our study. Third, our sample consisted of university students, limiting the generalizability of our findings to other populations. Future research should investigate these relationships in more diverse populations. Lastly, we did not control for potential confounding factors such as mental health conditions, which could influence both severity of migraine-like symptoms and executive functions. All participants provided symptom-based responses for MS-Q, which was used as a measure of migraine-like symptom severity in mediation analysis. However, to avoid misinterpretation, it is noteworthy to mention that severity estimates in participants below the screening threshold of 4 may not be reflective of clinical migraine. Future research should consider these factors to provide a more comprehensive understanding of the relationships observed in our study.

### Conclusion

Increasing severity of self-reported migraine symptoms was comorbid with (i) problems in attention and memory and (ii) problems in attention and fluid intelligence in university-attending young adults. Moreover, memory problems were comorbid with PFI unrelated to migraine severity.

## Data Availability

The data that support the findings of this study are available from the corresponding author, Dr. Mohammed F. Salahuddin, upon reasonable request.

## References

[ref1] Acosta GB , Wikinski SI , Bonelli CCG and Rubio MC (1996) Chronic administration of valproic acid induces a decrease in rat striatal glutamate and taurine levels. Amino Acids 10, 123–131. 24178474 10.1007/BF00806585

[ref2] Aggarwal M , Puri V and Puri S (2012) Serotonin and CGRP in migraine. Annals of Neurosciences 19(2), 88. 25205974 10.5214/ans.0972.7531.12190210 PMC4117050

[ref3] Agosti R (2018) Migraine burden of disease: from the patient’s experience to a socio-economic view. Headache: The Journal of Head and Face Pain 58(1), 17–32. 10.1111/head.13301 29697152

[ref4] Anaya F , Alia A , Hamoudeh F , Nazzal Z and Maraqa B (2022) Epidemiological and clinical characteristics of headache among medical students in Palestine: a cross sectional study. BMC Neurology 22(1), 1–8. 34979985 10.1186/s12883-021-02526-9 PMC8722108

[ref5] Appukuttan D , Vinayagavel M and Tadepalli A (2014) Utility and validity of a single-item visual analog scale for measuring dental anxiety in clinical practice. Journal of Oral Science 56(2), 151–156. 24930752 10.2334/josnusd.56.151

[ref7] Baron RM and Kenny DA (1986) The moderator–mediator variable distinction in social psychological research: conceptual, strategic, and statistical considerations. Journal of Personality and Social Psychology 51(6), 1173. 3806354 10.1037//0022-3514.51.6.1173

[ref8] Begasse de Dhaem O and Robbins MS (2022) Cognitive impairment in primary and secondary headache disorders. Current Pain and Headache Reports 26(1), 1–14. 35239156 10.1007/s11916-022-01039-5 PMC8891733

[ref9] Begum A , Dewey M , Hassiotis A , Prince M , Wessely S and Stewart R (2014) Subjective cognitive complaints across the adult life span: a 14-year analysis of trends and associations using the 1993, 2000 and 2007 English Psychiatric Morbidity Surveys. Psychological Medicine 44(9), 1977–1987. 24074262 10.1017/S0033291713002444

[ref10] Bigal ME , Bigal JM , Betti M , Bordini CA and Speciali JG (2001) Evaluation of the impact of migraine and episodic tension-type headache on the quality of life and performance of a university student population. Headache: The Journal of Head and Face Pain 41(7), 710–719. 10.1046/j.1526-4610.2001.041007710.x 11554960

[ref11] Braganza DL , Fitzpatrick LE , Nguyen ML and Crowe SF (2022) Interictal cognitive deficits in migraine sufferers: a meta-analysis. Neuropsychology Review 32(4), 736–757. 34398435 10.1007/s11065-021-09516-1

[ref12] Burgess PW and Simons JS (2005) Theories of frontal lobe executive function: clinical applications. In Halligan PW and Wade DT (eds), Effectiveness of Rehabilitation for Cognitive Deficits. New York: Oxford University Press, pp. 211–231.

[ref14] Buse DC , Rupnow MFT and Lipton RB (2009) Assessing and managing all aspects of migraine: migraine attacks, migraine-related functional impairment, common comorbidities, and quality of life. Mayo Clinic Proceedings 84(5), 422–435. 19411439 10.1016/S0025-6196(11)60561-2 PMC2676125

[ref15] Clemow DB , Johnson KW , Hochstetler HM , Ossipov MH , Hake AM and Blumenfeld AM (2020) Lasmiditan mechanism of action–review of a selective 5-HT 1F agonist. The Journal of Headache and Pain 21(1), 1–13. 32522164 10.1186/s10194-020-01132-3 PMC7288483

[ref16] Collins A and Koechlin E (2012) Reasoning, learning, and creativity: frontal lobe function and human decision-making. PLoS Biology 10, e1001293. 22479152 10.1371/journal.pbio.1001293 PMC3313946

[ref17] Comings DE (1994) Serotonin: a key to migraine disorders. Nutrition Health Review no. 70, 6. Gale Academic OneFile. Available at ===link.gale.com/apps/doc/A15911357/AONE?u=loyoland_main&sid=bookmark-AONE&xid=823df1fb (accessed 27 November 2023).

[ref18] Diamond A (2013) Executive functions. Annual Review of Psychology 64, 135–168. 10.1146/annurev-psych-113011-143750 PMC4084861 23020641

[ref19] Dong L , Dong W , Jin Y , Jiang Y , Li Z and Yu D (2025) The global burden of migraine: a 30-Year trend review and future projections by age, sex, country, and region. Pain and Therapy 14(1), 297–315. 39661241 10.1007/s40122-024-00690-7 PMC11751287

[ref20] Elisa RN , Balaguer-Ballester E and Parris BA (2016) Inattention, working memory, and goal neglect in a community sample. Frontiers in Psychology 7, 1428. 27713716 10.3389/fpsyg.2016.01428 PMC5031702

[ref21] Faedda N , Natalucci G , Calderoni D , Cerutti R , Verdecchia P and Guidetti V (2017) Metacognition and headache: which is the role in childhood and adolescence? Frontiers in Neurology 8, 650. 29312108 10.3389/fneur.2017.00650 PMC5735075

[ref22] Ferrer E , Shaywitz BA , Holahan JM , Marchione KE and Shaywitz SE (2009) Uncoupling of reading and IQ over time: empirical evidence for a definition of dyslexia. Psychological Science 21, 93–101. 20424029 10.1177/0956797609354084

[ref23] Flynn O , Fullen BM and Blake C (2023) Migraine in university students: a systematic review and meta-analysis. European Journal of Pain 27(1), 14–43. 36288401 10.1002/ejp.2047

[ref24] Gil-Gouveia R and Martins IP (2019) Cognition and cognitive impairment in migraine. Current Pain and Headache Reports 23(11), 1–10. 31511992 10.1007/s11916-019-0824-7

[ref27] Hansen TF , Hoeffding LK , Kogelman L , Haspang TM , Ullum H , Sørensen E , Erikstrup C , Pedersen OB , Nielsen KR and Hjalgrim H (2018) Comorbidity of migraine with ADHD in adults. BMC Neurology 18(1), 1–9. 30322380 10.1186/s12883-018-1149-6 PMC6190553

[ref28] Hayes AF (2013) The PROCESS macro for SPSS and SAS (version 2.13) [Software]. Available at https://www.processmacro.org/index.html (accessed 27 November 2023).

[ref29] Hayes AF and Coutts JJ (2020) Use omega rather than Cronbach’s alpha for estimating reliability. But… Communication Methods and Measures 14(1), 1–24.

[ref30] Kido-Nakahara M , Katoh N , Saeki H , Mizutani H , Hagihara A , Takeuchi S , Nakahara T , Masuda K , Tamagawa-Mineoka R and Nakagawa H (2015) Comparative cut-off value setting of pruritus intensity in visual analogue scale and verbal rating scale. Acta Dermato-Venereologica 95(3), 345–346. 25228475 10.2340/00015555-1972

[ref31] Kong Q , Currie N , Du K and Ruffman T (2022) General cognitive decline does not account for older adults’ worse emotion recognition and theory of mind. Scientific Reports 12(1), 1–11. 35473952 10.1038/s41598-022-10716-9 PMC9043191

[ref32] Láinez MJA , Domínguez M , Rejas J , Palacios G , Arriaza E , Garcia-Garcia M and Madrigal M (2005) Development and validation of the Migraine Screen Questionnaire (MS-Q) Headache: The Journal of Head and Face Pain 45(10), 1328–1338. 10.1111/j.1526-4610.2005.00265.x 16324165

[ref33] Láinez MJ , Castillo J , Domínguez M , Palacios G , Díaz S and Rejas J (2010) New uses of the Migraine Screen Questionnaire (MS-Q): validation in the Primary Care setting and ability to detect hidden migraine. MS-Q in Primary Care BMC Neurology 10(1), 1–8. 10.1186/1471-2377-10-39 PMC2906427 20529347

[ref34] Lauterbach EC , Shillcutt SD , Victoroff J , Coburn KL and Mendez MF (2010) Psychopharmacological neuroprotection in neurodegenerative disease: heuristic clinical applications. The Journal of Neuropsychiatry and Clinical Neurosciences 22(2), 130–154. 20463108 10.1176/jnp.2010.22.2.130

[ref35] Lehto JE , Juujärvi P , Kooistra L and Pulkkinen L (2003) Dimensions of executive functioning: evidence from children. British Journal of Developmental Psychology 21, 59–80.

[ref36] Le Pira F , Zappala G , Giuffrida S , Lo Bartolo ML , Reggio E , Morana R and Lanaia F (2000) Memory disturbances in migraine with and without aura: a strategy problem? Cephalalgia 20(5), 475–478. 11037744 10.1046/j.1468-2982.2000.00074.x

[ref37] Le Pira F , Reggio E , Quattrocchi G , Sanfilippo C , Maci T , Cavallaro T and Zappia M (2014) Executive dysfunctions in migraine with and without aura: what is the role of white matter lesions? Headache: The Journal of Head and Face Pain 54(1), 125–130. 10.1111/head.12158 23808818

[ref38] Manzar MD , Hameed UA , Salahuddin M , Khan MYA , Nureye D , Wakene W , Alamri M , Albougami A , PandiPerumal SR and Bahammam AS (2020) Migraine screen questionnaire: further psychometric evidence from categorical data methods. Health and Quality of Life Outcomes 18(1), 1–9. 32345313 10.1186/s12955-020-01361-9 PMC7189686

[ref41] May A and Schulte LH (2016) Chronic migraine: risk factors, mechanisms and treatment. Nature Reviews Neurology 12(8), 455–464. 27389092 10.1038/nrneurol.2016.93

[ref42] Mongini F , Keller R , Deregibus A , Barbalonga E and Mongini T (2005) Frontal lobe dysfunction in patients with chronic migraine: a clinical–neuropsychological study. Psychiatry Research 133(1), 101–106. 15698682 10.1016/j.psychres.2003.12.028

[ref43] Negro A , Koverech A and Martelletti P (2018) Serotonin receptor agonists in the acute treatment of migraine: a review on their therapeutic potential. Journal of Pain Research 1(1), 515–526. 10.2147/JPR.S132833 PMC5848843 29563831

[ref44] Olesen J and Lipton RB (1994) Migraine classification and diagnosis. International Headache Society criteria. Neurology 44, S6–10. 8008227

[ref45] Ossipov MH , Morimura K and Porreca F (2014) Descending pain modulation and chronification of pain. Current Opinion in Supportive and Palliative Care 8(2), 143. 24752199 10.1097/SPC.0000000000000055 PMC4301419

[ref46] Preacher KJ and Hayes AF (2004) SPSS and SAS procedures for estimating indirect effects in simple mediation models. Behavior Research Methods, Instruments and Computers 36(4), 717–731. 10.3758/bf03206553 15641418

[ref47] Reich A , Heisig M , Phan NQ , Taneda K , Takamori K , Takeuchi S , Furue M , Blome C , Augustin M and STäNDER S (2012) Visual analogue scale: evaluation of the instrument for the assessment of pruritus. Acta Dermato Venereologica 92(5), 497. 22102095 10.2340/00015555-1265

[ref48] Riva D , Aggio F , Vago C , Nichelli F , Andreucci E , Paruta N , D’Arrigo S , Pantaleoni C and Bulgheroni S (2006) Cognitive and behavioural effects of migraine in childhood and adolescence. Cephalalgia 26(5), 596–603. 16674769 10.1111/j.1468-2982.2006.01072.x

[ref49] Russo M , De Rosa MA , Calisi D , Consoli S , Evangelista G , Dono F , Santilli M , Granzotto A , Onofrj M and Sensi SL (2022) Migraine pharmacological treatment and cognitive impairment: risks and benefits. International Journal of Molecular Sciences 23(19), 11418. 36232720 10.3390/ijms231911418 PMC9569564

[ref50] Salem H , Vivas D , Cao F , Kazimi IF , Teixeira AL and Zeni CP (2018) ADHD is associated with migraine: a systematic review and meta-analysis. European Child & Adolescent Psychiatry 27(3), 267–277. 28905127 10.1007/s00787-017-1045-4

[ref51] SICUTERI F (1972) Headache as possible expression of deficiency of brain 5-hydroxytryptamine (central denervation supersensitivity). Headache: The Journal of Head and Face Pain 12(2), 69–72. 10.1111/j.1526-4610.1972.hed1202069.x 4262476

[ref52] Steiner TJ and Stovner LJ (2023) Global epidemiology of migraine and its implications for public health and health policy. Nature Reviews Neurology 19(1), 1–9. 36693999 10.1038/s41582-022-00763-1

[ref53] Švob Štrac D , Pivac N and Mück-Šeler D (2016) The serotonergic system and cognitive function. Translational Neuroscience 7(1), 35–49. 28123820 10.1515/tnsci-2016-0007 PMC5017596

[ref54] Vuralli D , Ayata C and Bolay H (2018) Cognitive dysfunction and migraine. The Journal of Headache and Pain 19(1), 1–14. 30442090 10.1186/s10194-018-0933-4 PMC6755588

[ref55] Zhang Y , Bi X , Adebiyi O , Wang J , Mooshekhian A , Cohen J , Wei Z , Wang F and Li X-M (2019) Venlafaxine improves the cognitive impairment and depression-like behaviors in a cuprizone mouse model by alleviating demyelination and neuroinflammation in the brain. Frontiers in Pharmacology 10, 332. 31024304 10.3389/fphar.2019.00332 PMC6460225

[ref56] Zhao X , Lynch Jr JG and Chen Q (2010) Reconsidering Baron and Kenny: myths and truths about mediation analysis. Journal of Consumer Research 37(2), 197–206.

